# The complete mitogenome of *Caridina indistincta* ‘sp. A’ (Decapoda: Atyidae)

**DOI:** 10.1080/23802359.2018.1467241

**Published:** 2018-08-16

**Authors:** Daniel J. Schmidt

**Affiliations:** Australian Rivers Institute, Griffith University, Nathan, Australia

**Keywords:** Freshwater shrimp, Atyidae, mitochondrial genome, MiSeq, genome skimming

## Abstract

A complete mitochondrial genome sequence was determined for a member of the *Caridina indistincta* species complex known as *C. indistincta* ‘sp. A’. The 15,461 bp sequence (GenBank: MH189850) was obtained via genome skimming, and contains 13 protein coding genes, 22 tRNA genes, 2 rRNA genes, and a 646 bp control region arranged in the pancrustacean ground pattern. *Caridina indistincta* sp. A is a freshwater macroinvertebrate important for ecosystem health monitoring in Australia and this reference will be a useful resource for metabarcoding and eDNA studies.

*Caridina indistincta* is a species complex of small freshwater atyid shrimps, widely distributed in eastern Australia (Page et al. 2005). The complex appears to consist of at least five species, informally known as *C. indistincta sp.* A, sp. B, sp. C, sp. D, and sp. E which can be morphologically diagnosed and are genetically divergent based on mtDNA (Page et al. 2005). The taxon *C. indistincta* sp. A is geographically restricted to 11 coastal basins spanning ∼350 km in southeastern Queensland, Australia, and is an indicator of biological condition in ecosystem health monitoring programs for this region (Page and Hughes 2007). A shallow whole-genome shotgun library of *C. indistincta* sp. A was prepared from a specimen collected in Mimosa Creek (27.54973 S, 153.05188 E) located on the Nathan Campus of Griffith University, Australia. Details of library preparation and sequencing (Illumina Miseq, insert size ∼500 bp) are reported elsewhere (Schmidt, Brockett, et al. 2016; Schmidt, Islam, et al. 2016). A total of 5.8 × 10^6^ reads were generated. Novoplasty v2.6.5 was used to assemble 3202 reads into a circular contig of 15,461 bp length with average coverage of ×82 (Dierckxsens et al. 2017). A seed sequence used to initiate assembly was retrieved using the mitogenome of *Caridina gracilipes* (NC_024751.1; Xu et al. 2016). The Mitos WebServer (Bernt et al. 2013) was used for initial gene annotation, followed by manual adjustment of gene boundaries after alignment with existing *Caridina* reference genomes (i.e. GenBank: KU726823; KM023648). The fully annotated new mitogenome is available at GenBank accession MH189850. No anomalies were detected – 13 protein coding genes, 22 tRNA genes, two rRNA genes and a 646 bp control region were arranged in the pancrustacean ground pattern which is common to other members of the Atyidae characterized to date (Tan et al. 2017). In a phylogenetic context, MH189850 was analysed with all available atyid mitogenomes on the NCBI RefSeq database, demonstrating its placement with other *Caridina* spp (Figure 1). A BLAST search (https://blast.ncbi.nlm.nih.gov/Blast.cgi) showed 100% identity between the new mitogenome and a 450 bp fragment of COI identified as *C. indistincta* sp. A, voucher GU84 sampled from the South Pine River, which is geographically proximate to the sampling location of the specimen analysed here (GenBank: JX913903; Page and Hughes 2014).

**Figure 1.**
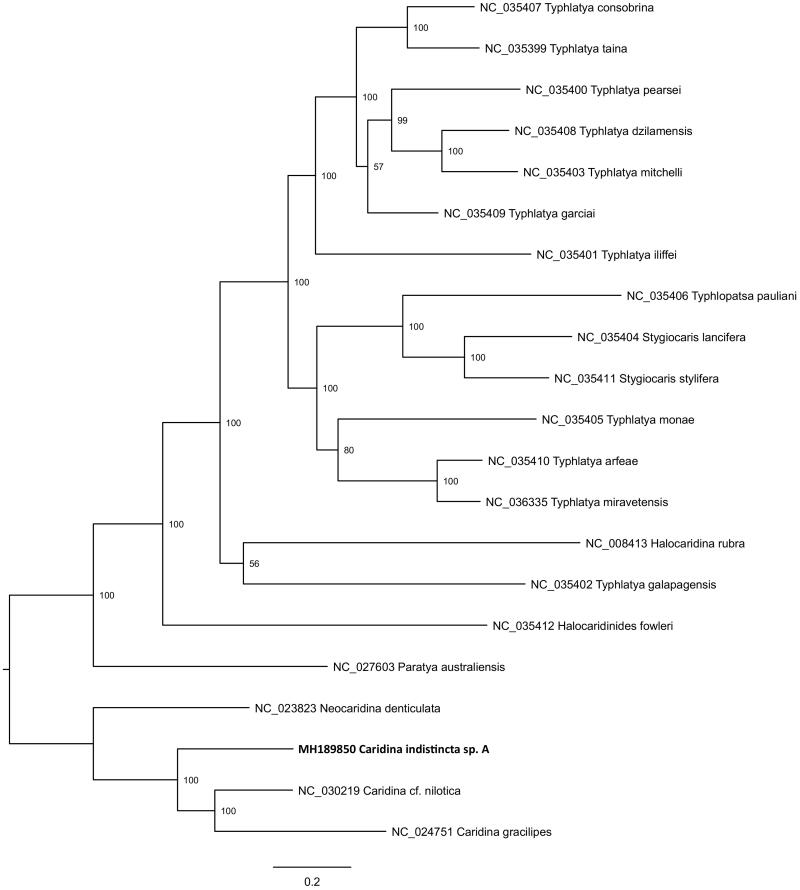
Phylogenetic placement of the new *Caridina indistincta* sp. A mitogenome (GenBank: MH189850) relative to 20 Atyidae mitogenomes sourced from the NCBI RefSeq database. Tip labels include GenBank accession and species name; node labels show bootstrap result. Alignment of mitogenomes (excluding 16S, 12S, and control region) was performed using MAFFT v7.017 (Katoh et al. 2002). A maximum likelihood phylogenetic analysis was performed on the final alignment of 12 821 bp with RAxML v8.2.11 using the GTR + GAMMA substitution model with 1000 bootstrap replicates (Stamatakis 2006).
